# Methylation-Demethylation Dynamics: Implications of Changes in Acute Kidney Injury

**DOI:** 10.1155/2018/8764384

**Published:** 2018-07-08

**Authors:** Anubhav Chakraborty, Pragasam Viswanathan

**Affiliations:** Renal Research Lab, Centre for Bio-Medical Research, School of Bio-Sciences and Technology, Vellore Institute of Technology, Vellore 632014, India

## Abstract

Over the years, the epigenetic landscape has grown increasingly complex. Until recently, methylation of DNA and histones was considered one of the most important epigenetic modifications. However, with the discovery of enzymes involved in the demethylation process, several exciting prospects have emerged that focus on the dynamic regulation of methylation and its crucial role in development and disease. An interplay of the methylation-demethylation machinery controls the process of gene expression. Since acute kidney injury (AKI), a major risk factor for chronic kidney disease and death, is characterised by aberrant expression of genes, understanding the dynamics of methylation and demethylation will provide new insights into the intricacies of the disease. Research on epigenetics in AKI has only made its mark in the recent years but has provided compelling evidence that implicates the involvement of methylation and demethylation changes in its pathophysiology. In this review, we explore the role of methylation and demethylation machinery in cellular epigenetic control and further discuss the contribution of methylomic changes and histone modifications to the pathophysiology of AKI.

## 1. Introduction

Epigenetic mechanisms effectuate a broad variety of gene expression in several cells and tissues of multicellular organisms. Epigenetics refers to the modification and modulation of gene expression without imparting any direct alteration in the DNA sequence. DNA methylation is one such epigenetic mark that exists to control a variety of gene expression in eukaryotes. The first evidence of DNA methylation was observed as modified cytosine demonstrated by Rollin Hotchkiss in 1948 using paper chromatography. It was only in the 1980s that studies started demonstrating the role of DNA methylation in controlling gene expression [1, 2], and now, decades of research have successfully established its importance in growth, development, and diseases. Along with enzymes catalysing DNA methylation, there also exists “erasers” that remove DNA methylation. This process of modifying or removing methyl groups is epigenetically termed as DNA demethylation. With accumulating evidence of molecules and mechanisms in reversing DNA methylation in mammalian cells, it has become evident that the interplay between methylation and demethylation severs to control or maintain a stable cellular functionality [3].

With recent advances in epigenetic technology and understanding of the mammalian methylome, several epigenetic modifications have been implicated in the pathogenesis of acute kidney injury (AKI). AKI is characterised by rapid fall in renal function and crude renal structural changes [4] with an increased risk of chronic kidney diseases and end-stage renal failure [5]. The pathophysiology of AKI as a result of insults such as ischemia-reperfusion, sepsis, contrast media, rhabdomylosis, and nephrotoxins is well documented [6, 7]. The clinical causes of AKI are grouped as prerenal, renal, and postrenal [8], but with the increased number of studies with ischemic and nephrotoxic insults, it is considered that renal/intrinsic factors are mostly associated with actual renal tissue injury. Of 35–70% of AKI as a result of renal etiologies; ischemic injury or nephrotoxins contribute to 80–90% of the intrinsic factor [9]. Although the cellular and molecular abnormalities during AKI have been vividly investigated, the epigenetic understanding of AKI onset, progression, and treatment is still at its nascent stage. Upon understanding the importance of epigenetic processes in other model systems, it is most likely that epigenetics plays a crucial part in AKI [10–13]. Although DNA methylation is the best characterised epigenetic mark, it is yet to be intensively described in the context of renal pathology. The discovery of 5-hydroxymethylcytosine (5hmC) has brought interesting findings and opinions on the essential role of DNA methylation and demethylation in development and diseases. The bulk of research on 5hmc has, however, been focussing on neural and cancer studies, but with recent advances in global, locus-specific, and single nucleotide 5hmc analysis, it will be exciting to understand the role and mechanism of action of 5hmc in the pathological conditions of the kidney.

In this review, we introduce the readers to a comprehensive understanding of the DNA methylation and demethylation machinery and their role in epigenetic regulation of gene and cellular mechanisms. We further highlight the advances in epigenetic regulation of AKI by providing insight into the implications of methylomic changes and histone modifications in the pathogenesis of AKI.

## 2. DNA Methylation

DNA methylation represents a mechanism of cellular memory. This was first proposed independently in 1975 by Holliday and Pugh [1] as well as Riggs [14]. Both groups hypothesised that CpG methylation and nonmethylation patterns could be copied during cell division. This was originally based on the fact that cytosine methylation in mammalian cells occurs predominantly in CpG dinucleotides. Once the DNA replicates, the parental DNA strand would maintain its pattern of modified cytosines while the newly synthesized strand remains unmodified. To establish proper copying of the parental pattern onto the progeny strand, both these groups postulated an enzyme that would methylate CpGs base-paired with a methylated parental CpG. This was the task of methyltransferases. The action of methyltransferases would result in patterns of DNA methylation that would replicate semiconservatively like the base sequence of DNA. DNA methylation in mammals refers to the transfer of the methyl group from S-adenosyl methionine (SAM-CH3) to cytosines in the CpG dinucleotides [15] contributing to epigenetic inheritance and has a vital role in development and diseases. Methylated cytosines or 5mC is found almost entirely within CpG dinucleotides [16]. The genome of mammalian somatic tissues is methylated at 70%–80% of all CpG dyads. Often satellite DNAs, repetitive elements, nonrepetitive intergenic DNA, and exons of genes bear high levels of methylation. However, CpG islands are an exception to this global methylation as they remain mostly unmethylated in the germline, the early embryos, and also in most somatic tissues [17]. CpG clusters called CpG islands are most often situated in gene promoters and regions more toward the 3′ end [18]. They function as strong promoters and also as replication origins [19]. Contrary to the popular understanding that CpG islands are known to be mostly unmethylated [17], during the embryogenic process of X chromosome inactivation in female placental mammals, CpG islands become de novo methylated [20] leading to silencing of genes on the inactivated chromosome necessary for dosage compensation [21]. Apart from X chromosome inactivation, DNA methylation plays a crucial role in suppressing retrotransposon elements, genomic imprinting [16], and for normal development [22, 23]. We now know that DNA methylation also occurs at non-CpG sites such as CpA, CpT, and CpC. [24]. An interesting feature of non-CpG methylation is that it is most frequently found at CpA dinucleotide, and several studies using whole genome bisulphite sequencing have described the trend in frequency of methylation at each dinucleotide (CpG > CpA > CpT > CpC) [25–28]. Also, non-CpG dinucleotides represent an asymmetrical sequence, for example, CpA dinucleotides are paired with complimentary TpG dinucleotides on the opposite strand (CpA:TpG). Therefore, non-CpG methylation is often only present on one DNA strand at any given site [24]. It is, however, not the same in case of the palindromic CpG dinucleotides, which are usually symmetrical (CpG:GpC) leading to methylation at cytosine residues on both DNA strands [24]. Few studies show that non-CpG methylation is only a by-product of the hyperactivity of nonspecific de novo methylation of CpG sites [27, 29]. However, other reports argue that non-CpG methylation is correlated with gene expression and tissue specificity [25, 30, 31]. Although there is now evidence of non-CpG methylation occurring within various cell types and specific tissues, its functional relevance in the mammalian genome is yet to be completely understood.

## 3. Patterns of DNA Methylation

**Figure 1:**
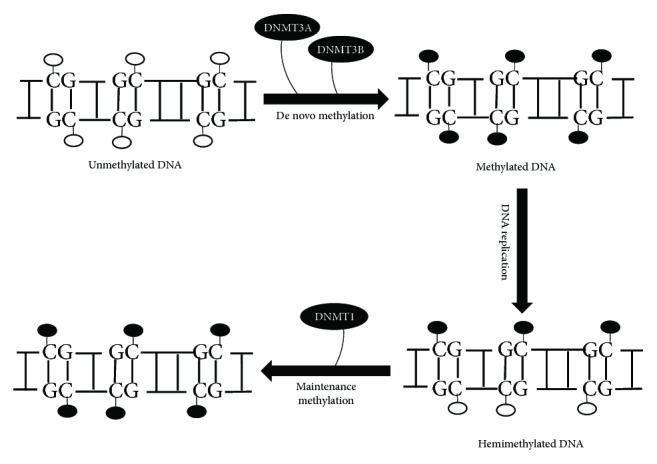
De novo DNA methylation and maintenance. Methylation of unmethylated DNA (shown as white circles) occurs when a methyl group is transferred to the cytosine residue in the DNA, mainly in the CpG dinucleotide. The de novo methyltransferases (DNMT3a and DNMT3b) catalyse this process thereby generating 5-methylcytosine (shown as black circles), and upon DNA replication, the DNMT1 acts on the hemimethylated DNA to maintain the fidelity of inherited methylation patterns.

DNA methylation pattern is highly tissue-specific, nonrandom, and well maintained [32, 33]. There exist two models accounting for the methylation pattern in mammals: (i) proteins bound to specific DNA regions leads to inaccessibility to methylation sites [34, 35] and (ii) proteins bound to specific DNA sequence directing the methylation targeting mechanism [36–38]. The first model is referred as the exclusion model where studies reported by Macleod et al. [34] and Brandeis et al. [35] showed that when Sp1 binding sites flanking a CpG island are removed, it led to the de novo methylation of the CpG island during development. Studies by Macleod et al. [34] and Brandeis et al. [35] suggest that when Sp1 sites flanking the CpG islands are occupied by Sp1 transcription factors, it causes the DNA methylases to not gain access to the relevant CpG island. Also, the methylation state of the sites to which DNA-binding protein binds can also be affected if there is any modulation in the affinity of the DNA binding [39, 40]. As a result of these experiments, it can be suggested that exclusion of de novo methyltransferases (DNMTs) might have a role in the formation of patterns such as those that occur in early development [41]. Although the targeting model has arisen from the association of DNMTs with certain proteins (specifically with proteins including Rb, E2F1, histone deacetylases (HDACs), and the transcriptional repressor RP5), there is no evidence of such interactions leading to de novo methylation. Earlier, it was believed based on experimental evidence that the maintenance methylation model drives the patterning process. Here, the de novo methyltransferases DNMT3a and DNMT3b were thought to establish the methylation patterns at the early development which is further maintained through somatic cell divisions by maintenance methyltransferase DNMT1, acting on the hemimethylated CpG sites generated by DNA replication (Figure 1) [1, 14]. In the case of non-CpG methylation, the role of only DNMT3a and DNMT3b is attributable whereas DNMT1 is not associated with non-CpG methylation patterns [42, 43]. *In vivo* studies showed that DNMT1 knockout mouse embryonic stem (ES) cells retained patterns of non-CpG methylation [42] whereas DNMT3l (a regulatory molecule) knockout mice had significantly lower levels of CpA methylation in their prospermatogonia [44]. Furthermore, DNMT3a and DNMT3b double knockout ES cells were reported to have much lower CpA methylation levels [43, 45]. Therefore, unlike CpG methylation, non-CpG methylation would need to be reestablished de novo after each cell division in order to be maintained. This claim is supported by the study of Ichiyanagi et al. [44] that non-CpG methylation accumulates in nondividing male mouse germ cells but is rapidly lost following the recommencement of cell division. Most changes in methylation patterns occur during mammalian development and cell differentiation. In mouse germ cells and early embryos, there occur large scale demethylation and remethylation changes [46]. Once fertilization occurs, the paternal genome undergoes rapid demethylation [47, 48] and the maternal genome undergoes passive, replication-dependent demethylation during subsequent cleavage divisions [48]. Once implantation occurs, global de novo methylation takes place that reestablishes the DNA methylation patterns which are maintained in somatic tissues. Another example of dynamic methylation patterning could be seen during lineage-specific differentiation of hematopoietic progenitors when gene-specific de novo methylation and demethylation occur [49].

## 4. De Novo Methylation by De Novo Methyltransferases

DNMT3a and DNMT3b are the DNA methyltransferases playing a role in the process of mammalian DNA methylation. DNMT2 is another candidate methyltransferase that has limited DNA methyltransferase activity *in vitro*, and lack of DNMT2 has no effect on de novo or maintenance methylation of DNA [50]. In fact, DNMT2 has been shown to specifically methylate cytosine 38 in the anticodon loop of transfer RNA^Asp^ [51]. DNMT3a and 3b are structurally related and do not require hemimethylated DNA to bind; they show an equal affinity for hemimethylated and unmethylated DNA [52]. However, both DNMT3a and 3b are highly required for de novo methylation as evidenced from study in the ES cells and embryos where the absence of DNMT3a and 3b led to exclusion of de novo methylation of proviral genomes and repetitive elements [22]. Unlike DNMT3a which is expressed ubiquitously, DNMT3b is poorly expressed by the majority of differentiated tissues except for thyroid, testes, and bone marrow [53]. Also, DNMT3a in association with DNMT3l, a regulatory molecule, is required for establishing distinct DNA methylation patterns on imprinted genes [54].

Structural studies have shown that the C-terminal domains of DNMT3a and 3l form a tetrameric complex (3l-3a-3a-3l) with two active sites [55] that can methylate two CpGs spaced by 8–10 bp, *in vitro* [56, 57]. Similar to DNMT3l, there are other interacting factors involved in de novo methylation at specific genomic regions. For example, the Piwi-interacting small RNA pathway plays a vital role in de novo methylation of retrotransposons in fetal male germ cells. However, its mechanism is yet to be elucidated [58, 59]. DNMT3a and 3b are also responsible for establishing methylation patterns during early development [22]. De novo DNA methylation was first described in an experiment where foreign DNA became methylated upon introduction into a preimplantation embryo in an unmethylated state. A study by Jähner et al. [60] reported stable retroviral DNA methylation from infected mouse preimplantation embryos and also a stable methylated DNA injected into mouse zygotes; however, no retroviral DNA methylation occurred in embryos at the later stage of gastrulation. This study supports de novo methylation in the early embryonic stage and suggests that this process is confined to the pluripotent cells of early embryos. Additional support of this theory was provided by Stewart et al. [61] when they reported that in retroviral infected cells, the retroviral DNA was completely methylated and the viral genes were silenced. The same authors gave another indication of de novo methylation occurring in early development by reporting that somatic cells infected with viral DNA did not undergo any methylation.

Although much is known about the enzymes partaking in the de novo methylation process, not much is known as to how de novo DNMTs target specific genetic regions. Recent structural and biochemical studies showed that the plant homeodomain (PHD) of DNMT3a could directly interact with H3 tails unmethylated at Lys-4 *in vitro* [62–64]. Also, the PWWP domain located in the N-terminal parts of DNMT3a and 3b can interact with H3 tails with trimethylated Lys-36 (H3K36me3) *in vitro* [65]. Another theory is that DNMTs are recruited to specific sequence motifs by transcription factors that bind to the DNA thereby catalysing DNA methylation or preventing it. DNMTs may also bind to transcription factors to target DNA methylation [66]. In case of a mutation in the transcription factor binding site spanning the CpG islands, these regions are unable to retain their unmethylated state [34, 35], thereby implicating that exposed CpG islands serve as direct targets of DNMTs. Based on these proposed mechanisms, it can be suggested that either de novo methylation by DNMT3a and 3b occurs by their recruitment to gene promoters by specific transcription factors or they may perform genome-wide methylation of all unprotected CpG sites.

## 5. DNA Methylation and Gene Expression

Methylation of promoters leads to transcriptional repression [67]. DNA methylation studies in mammalian cells came into light when Jones and Taylor in 1980 described that 5-azacytidine, a nucleoside analog, was capable of inhibiting DNA methylation in living cells [68]. The concept that DNA methylation leads to gene repression was further supported by genetic analysis using DNMT1 knockout mice [69]. Li et al. [69] showed that when DNMT1 is inactivated, there occurs a genome-wide loss of DNA methylation and activation of X silenced genes, viral genes, and imprinted genes like H19 and IGF2. Several transcription factors bind to GC-rich sequence motifs that can contain CpG sequences. However, when CpGs are methylated, the binding of these transcription factors is severely hindered [70]. Bell and Felsenfeld in their study [71] showed that the CpG-rich CTCF binding sites are methylated at the paternal locus thereby preventing CTCF binding and thereby allowing the downstream enhancer to activate Igf2 expression. In the maternally derived copy of the Igf2 locus, the gene is silent as CTCF binds between its promoter and a downstream enhancer. This study describes the role of DNA methylation in gene regulation by regulating the transcriptional process.

DNA binding factors can also interfere with DNA methylation patterns. Stalder et al. [72] showed that embryonic stem cells and neuronal progenitors consisted of distal regulatory regions with poor CpG and with a low level of methylation. These were termed as low methylated regions, and the binding of transcription factors in these regions was indications of local DNA methylation being influenced by their binding. Another mode of gene repression by DNA methylation is by recruiting proteins to methyl-CpG regions. Methyl-CpG-binding protein complexes (MeCP1 and MeCP2) were the initial proteins identified to take part in this mechanism [73]. Later, the methyl-CpG-binding domain protein (MBD) family was identified comprising MeCP2, MBD1, MBD2, MBD3, and MBD4 (Table 1) [74]. Bird and Wolffe [74] described that MBD1, MBD2, and MeCP2 were key players in methylation-dependent repression of transcription. During DNA replication, silencing of genes by MBD1 occurs when it associates with histone lysine methyltransferase SETDB1 [75] leading to continuous H3K9 methylation at the target sequence. The DNA-binding component of MeCP1 is MBD2 [76, 77]. A study by Hendrich et al. [78] showed that MBD2-deficient mice have aberrations in tissue-specific gene expression. MeCP2 together with mSin3a corepressor complex depends on histone deacetylation for its repressive action [79, 80], and these findings show that each of the four methyl-CpG-binding proteins associates with different corepressor complexes for carrying out their action.

Few important studies have also provided compelling evidence of non-CpG methylation having a functional role in mammalian gene expression. Malone et al. [81] showed that when the Cp^m^CpNpGpG site within the promoter of the B29 gene is methylated, it represses the promoter activity in human B cells by blocking the binding of the early B cell factor, a transcription factor. Also, within the human SYT11 promoter region, non-CpG methylation of the Sp transcription factor binding sites reduces the binding of Sp proteins and associated transcription factors [82]. Barrès et al. [30] showed that the promoter of the peroxisome proliferator-activated receptor-*γ* coactivator 1*α* (PGC-1*α*) gene is more methylated at non-CpG sites in patients with type 2 diabetes mellitus and it results in the downregulation of PGC-1*α*. Transcriptional repression in brain cells because of non-CpG methylation has also been reported [83]. Bellizzi et al. [84] in their study of mitochondrial DNA methylation has reported that the D-loop region which contributes to the regulation of mitochondrial DNA replication and transcription has ~50% of methylcytosine within non-CpG dinucleotides. Collectively, these studies implicate that non-CpG methylation at gene promoters is associated with reduced gene expression.

## 6. Methylation and Chromatin Changes

Inside the nuclei of eukaryotic cells, there exists a complex of DNA and proteins, together forming the chromatin. The nucleosome is the fundamental repeating unit of chromatin characterised by DNA of length 146 base pairs wrapped around the histone protein cores (two H2A/H2B dimers and an H3/H4 tetramer). Methylation of these histone proteins changes the transcription machinery by providing certain proteins (chromatin modifiers) with docking sites, thereby creating an active or repressive chromatin structure and transcriptional marks. Histone H3 lysine residues (K4, K9, K23, K27, K36, K56, and K79), K20 in H4, K26 in H1, and arginine (R) residues (R2, R8, R17, and R26) in H3 along with R11, R12 in H2A, and R3 in H4 are the known methylation sites [88, 89]. This methylation is mediated by histone methyltransferases namely lysine-specific (KMT) and arginine-specific (RMT). Except H3K79, located within the nucleosome core, all other sites are found within the histone tails. Modifications of both histone tails and residues within the core play a role in gene expression [90].

Amidst the known methylated sites in histone proteins, one of the best studied methylated histone marks is H3K4 which is known to protect DNA from de novo methylation [91, 92]. The PHD-like domain of DNMT3l initiates de novo methylation by recruiting DNMT3a2 (germ cell-specific isoform of DNMT3a) to the nucleosomes that contain unmethylated H3K4, and this interaction gets abolished if H3K4 is methylated [57, 93, 94]. This inverse correlation between H3K4 methylation and DNA methylation suggests that in certain imprinted regions in germ cells, it is crucial that H3K4 undergoes demethylation for de novo methylation to take place. Furthermore, biochemical and structural studies revealed that the ATRX-DNMT3-DNMT3l (ADD) domain found in DNMT3a and DNMT3b could directly interact with H3 tails unmethylated at lysine 4 *in vitro* even without any accessory proteins [63, 64, 95]. Also, the PWWP domain of DNMT3a specifically interacts with H3K36me3 *in vitro* [65]. This interaction leads to an increase in activity of DNMT3a2 on chromatin-bound DNA [64, 65]. Genome-wide studies have found that H3K36me3 is located mainly in the bodies of active genes [96, 97] and this modification positively correlates with DNA methylation, therefore, suggesting that DNMT3a can specifically recognize histone modifications and then methylate the associated DNA [96, 97]. The *in vivo* evidence of the interactions of DNMTs with histone tails has been reported in several studies [99, 100]. Noh et al. [99] in their study demonstrated that DNMT3A enzyme with an engineered ADD domain is able to bind to H3K4me3 and H3T4 phosphorylated within the H3 N terminus (H3T4ph). Morselli et al. [100] showed that in *Saccharomyces cerevisiae* that expressed a heterologous DNMT3b or in mouse ES cells, DNMT3b is specifically enriched at H3K36me3 sites and that recruitment is based on binding affinity. DNA methylation in mammals is also regulated by histone H3K9 methyltransferases (G9a and Suv39h). Few major satellite repeats, retrotransposons, and the G9a promoter were found to be significantly lacking DNA methylation in G9a-negative mouse ES cells [101, 102]. However, G9a^−^/^−^ cells expressing a G9a mutant that is defective in the methylation of H3K9 were found to undergo normal DNA methylation suggesting that DNA methylation does not require methylation of H3K9 [101–103]. It is believed that G9a can recruit DNMT3 methyltransferases to the promoters for de novo methylation upon interacting with DNMT3a and DNMT3b [103]. Likewise, double knock out of Suv39h1 and Suv39h2 in mouse ES cells was seen to result in loss of methylation at pericentric major satellite repeats [104]; however, their way of contributing to DNA methylation is still unclear. Most euchromatin regions are characterised by H3K79 methylation [105] mediated by DOT1L, a histone H3K79 methyltransferase [106], and most of the transcriptionally repressed genes are enriched in H3K9m2, H3K9m3, H3K27m2, H3K27m3, H4K20m2, and H4K20m3 [107].

## 7. DNA Demethylation

**Figure 2:**
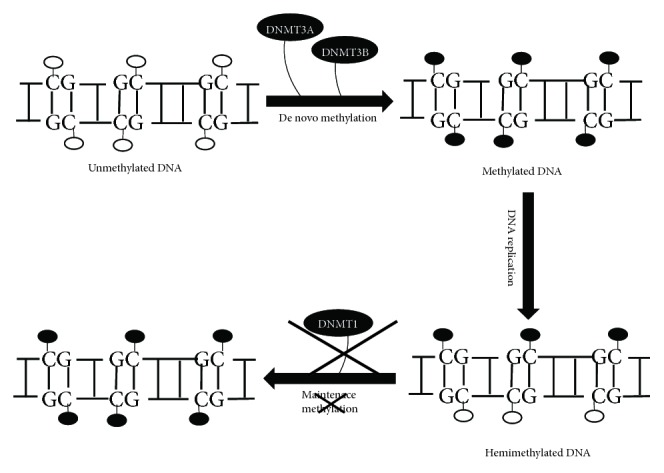
Mechanism of passive DNA demethylation. De novo methyltransferase 1 (DNMT1) is required to maintain the methylation pattern through successive replication; however, during reduced activity or in the absence of the maintenance DNA methyltransferase (DNMT1), there occurs progressive dilution of 5-methylcytosine (5mC) leading to the formation of unmethylated DNA.

**Figure 3:**
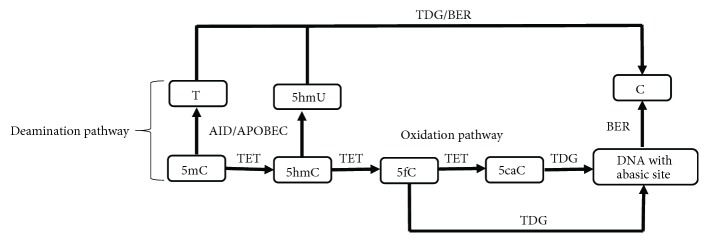
Mechanism of active DNA demethylation. The active process of DNA demethylation progresses either through the deamination or the oxidation pathway. In the case of the deamination pathway, the cytosine (C) residue of 5-methylcytosine (5mC) and 5-hydroxymethylcytosine (5hmC) undergoes deamination catalysed by AID/APOBEC enzymes to generate 5-hydroxymethyluracil (5hmU) and thymine (T) bases which are replaced by C during base excision repair (BER) mediated by thymine DNA glycosylase (TDG). The oxidation pathway mediated by the ten-eleven translocation (TET) family of enzymes can hydroxylate 5mC to form 5hmC which upon oxidation forms 5fC and 5caC. These oxidation products (5fC and 5caC) can be removed by TDG to generate an abasic site which is repaired by the BER pathway to generate a cytosine.

Although the underlying molecular mechanism of DNA demethylation is still unclear, several accumulating evidences suggest that the process of DNA methylation is reversible. The process of reversal is either by active replacement of methylated cytosine residues to unmodified cytosine or by passive demethylation during cell division due to inactive DNA methyltransferases (DNMTs) [108–110]. A classic example of passive DNA demethylation in mammalian development is the replication-dependent process of dilution of methylation marks in the maternal genome during preimplantation growth [48]. The passive process occurs simply by not methylating the new DNA strand after replication as a result of reduced activity or the absence of DNMTs (Figure 2) [111]. The active process of genome-wide DNA demethylation is well studied in zygotes [47, 48], in primordial germ cells [46, 112], and T lymphocytes [113]. Also, locus-specific active demethylation has been evidenced in somatic cells, such as neurons [114]. Although there exists numerous concrete evidences of active DNA demethylation, the mechanisms remain poorly understood. The mechanisms of enzymatic removal of the 5-methyl group from the modified cytosine residue involve several players of the active demethylation pathway (Figure 3). Also, the mechanism(s) of locus-specific demethylation might be different from the global genome-wide demethylation process. However, the process, the conversion of the methylated cytosine to an unmodified base, is unlikely to be a one-step process. Unlike plants, there is no known mammalian homolog for the DME/ROS1 family of 5mC-specific DNA glycosylase that can directly remove the 5mC base. Thus, all 5-methylcytosine (5mC) to the C conversion process known so far involves modifying the base either by oxidation or deamination followed by replacement of the modified base [108, 115].

## 8. Mammalian Glycosylases in Active DNA Demethylation

In *Arabidopsis*, the DME/ROS1 family of 5mC DNA glycosylases had been shown to perform active demethylation of specific genes [115]. For example, ROS1 with its apurinic/apyrimidinic lyase activity first removes the methylated base and then cleaves the basic site, leaving a nick, which then gets repaired [115]. This process resembles the base excision repair (BER) mechanism in mammals; however, the enzyme and the underlying process appear to be different from those in *Arabidopsis*. With no mammalian homolog of the DME/ROS1 family, it was earlier believed that thymine DNA glycosylase (TDG) and MBD4 might exert DNA demethylating activity [116, 117]. However, for both these glycosylases, the activity toward T-G mismatch was higher than 5mC and was, therefore, considered weak 5mC glycosylases [116, 117].

## 9. DNA Demethylation by the AID/APOBEC Family

Activation-induced deaminase (AID) has been a subject of the intense study over several years because of its critical role in hypermutation, class switch recombination, and gene conversion in activated B cells [118, 119]. It was only recently that the role of AID in DNA demethylation was reported [120, 121]. The AID is a member of the apolipoprotein B mRNA-editing catalytic polypeptide (APOBEC) family [122] and was identified in an experimental screening of cytosine deaminases expressed in mouse oocytes [123]. AID deaminates cytosine residues to uracil, which are then repaired by either base excision (BER) or mismatch repair (MMR) [124, 125]. The role of AID in global DNA demethylation was first discovered in zebrafish embryos, and the study indicated that the overexpression of both AID and MBD 4, but not either alone, was required for demethylation of DNA [126]. Similar experimental evidence was also given by Popp and colleagues who suggested a role of AID in global DNA demethylation at a later stage of mice embryogenesis [127]. Studies with AID null mice also showed increased DNA hypermethylation in primordial germ cells suggesting the role of AID in DNA demethylation [119]. Nuclear reprogramming studies provided the first evidence suggesting the role of AID in DNA demethylation in mammals and somatic cells [120]. Two independent groups working on nondividing heterokaryons (fusion of mouse ES cells with human fibroblast cells) reported that AID knockdown reduced reprogramming efficiency and also impaired demethylation of promoters of genes (OCT4 and NANOG) associated with pluripotency [120, 128]. With several studies addressing the role of AID in DNA demethylation, there is a considerable uprise in conflicting results. The role of AID in deamination in the DNA demethylation process in mammals is yet to reach a consensus conclusion. In a study of methylation dynamics of mouse germinal center B cells, the authors found no DNA demethylating effect of AID when using cells in culture [129]. DNA methylation was also found to be slightly higher in primordial germ cells of AID null mice compared to that of controls [127] although the difference in the genetic background of AID null and control mice might have influenced the result [130]. In another study, demethylation as a result of deamination was suggested based on the detection of a complex of Tdg, Gadd45a, and AID, but the activity of AID on 5mC containing DNA was not directly demonstrated [121]. It was also suggested that AID might deaminate C and not 5mC [131–133]. This indirect deamination leads to the replacement of 5mC by unmethylated C in the vicinity [134–136]. The conflicting data on the role of AID necessitates future studies of its action and also its role in active DNA demethylation.

## 10. Implications of TETs in Oxidative Demethylation of DNA

Ten-eleven translocation (TET) proteins are Fe(II)-dependent dioxygenases constituting the human TET1, TET2, and TET3 enzymes [137]. Although Wyatt and Cohen in 1952 [138] reported the existence of 5-hydroxymethylcytosine (5hmC), it was not until Tahiliani et al. [139] who identified these three human TET family proteins and showed that TET1 can catalyse conversion of 5mC to 5hmC *in vitro* and in cultured cells that its significance came to be known. The TET family enzymes work by splitting oxygen molecule into its constituent atoms. The splitting of molecular oxygen occurs while bound to the iron in the active site of TET, and this reaction catalyses the oxidation of the DNA base. After the split, one oxygen atom inserts into the 5-substituent of the cytosine base, converting 5mc to 5hmc. TET enzymes function to convert 5mC to 5hmC [139, 140], 5hmC to 5-formylcytosine (5fC), and 5fC to 5-carboxylcytosine (5caC) [137]. The oxidation of 5mC by TETs reduces the levels of 5mC, and a loss of TETs causes hypermethylation [141]. TET paralogs were also found in zebrafish and mouse, and all three mouse TET proteins are known to catalyse a similar reaction [142]. While extending and supporting the report of Tahiliani et al. [139] in describing the role of TET1 in regulating DNA methylation, Shinsuke et al. [142] in their study in mouse ES cells found that TET1 was required for keeping the NANOG promoter in a hypomethylated state. However, there are alternate suggestions where these proteins are sought to mediate the regulation of lineage-specific genes and not NANOG studies despite their high levels in ES cells [143, 144].

After the discovery of 5hmC, it was believed that TETs could remove the repression of gene expression caused by 5mC at several gene promoters [139, 142]. However, reports of nonproductive transcription and inactive genes even in the presence of high concentration of 5hmC [143, 145–147] contradict the popular belief and suggest that 5mC to 5hmC conversion is not functionally similar to the conversion of 5mC to C [145]. In two independent studies, one with triple TET knockout ES cells [141] and the other with murine embryonic fibroblasts (MEFs) [148], it was found that these cells were viable but were defective in their ability of differentiating and dedifferentiating. The dedifferentiation defect of MEFs was a result of an impaired mesenchymal-to-epithelial transition (MET) and could be reversed by overexpression of miRNAs that were initially suppressed due to TET deficiency [141, 148]. In one study, TET overexpression was seen to enable reprogramming where TET1 along with Oct4 led to induced pluripotent stem cells (iPSCs) while other exogenous reprogramming factors were absent [149]. These effects are most likely mediated by the control of the methylation state of enhancers [150, 151]. Other effects of TET deletion can be seen as partly penetrant midgestation abnormalities due to TET1 and TET2 double knockout [152], gastrulation defects due to the decrease in expression of the *Lefty* genes that antagonize Nodal signalling leading to Nodal gain [153]. TET3 deficiency in *Xenopus* has been studied as a causative to the eye and neural developmental defects [154]. A tripe TET gene ablation in zebrafish leads to death beyond the larval stage [155]. Studies in developmental genetics have found that prior to cell division, TET3 plays a major role in the active loss of 5mC in male pronucleus upon zygote formation. This rapid loss of 5mC was due to an increase in the 5hmC level implicating 5mC to 5hmC conversion [156, 157]. Upon knocking out TET3 in mouse zygotes by RNA interference (RNAi), there was an increase in the 5mC level suggesting a huge potential for TET proteins in DNA demethylation in early development. TET may also function cooperatively with AID in DNA demethylation activity [121] with AID acting upstream or downstream of TETs but not on the same base. However, such claims require additional experiments that would probe into DNA methylation and demethylation mechanisms and their regulation.

## 11. Base Excision Repair Mechanism by the BER Glycosylase Family in DNA Demethylation

Active demethylation requires cytosine replacement via DNA repair. Unlike plants, the base excision repair (BER) mechanism in mammals is quite complex as there are no glycosylases identified so far that would act directly on 5mC or 5hmC. An intermediate step of deamination precedes the BER mechanism in mammals [121, 158]. Uracil generated after cytosine deamination and upon mispairing with guanine is excised by uracil-DNA glycosylase 2 (UDG2), single-strand-selective monofunctional uracil-DNA glycosylase 1 (SMUG1), MBD4, and thymine-DNA glycosylase (TDG) [159]. Also, deamination of 5mC leading to the formation of thymine upon mispairing with guanine is excised by TDG and MBD4. UDG2 [135] and TDG [136] have also been reported to remove 5mC bases in the vicinity by either canonical mismatch repair or by long patch base excision. These enzymes constitute the family of glycosylases implicated in the BER pathway. Unlike deaminated methylcytosine, oxidised methylcytosine derivatives do not undergo mispairing [160, 161]; however, 5caC-G pairs may resemble T-G mispairs [161, 162] and are known to have weak base pairing [163]. Both 5fC and 5caC have weak glycosidic bonds [162, 163] and therefore, resemble BER substrates for excision. Uracil-DNA glycosylase (UDG) occurs as UDG2 in nuclear isoform as a result of alternate splicing and is capable of removing uracil generated by deamination of cytosine. Although it was initially found that *in vitro* UDG2 possessed no activity against substrates containing 5caC [164], later experiments contradicted this finding by reporting an active form of UDG2 in cultured cells that prevented the accumulation of 5caC in genomic DNA resulting from TET2 (catalytic domain) overexpression [165]. High levels of UDG expression in the zygote and early embryos led to a proposition of its role in DNA demethylation at this stage. Zygote deficient of UDG was found to have impaired demethylation at selected loci, specifically, NANOG and LINE-1 elements [165]. Studies have also reported a cooperative role of UDG2 with AID in active DNA demethylation in zygote [135]. It is, however, unclear if it is deamination or oxidation-based demethylation that is affected by a loss of UDG.

Study in UDG-deficient mice has shown that despite elevated levels of uracil in DNA, the animals develop naturally and show no unconcealed phenotype [166]. TDG was the first reported enzyme with the ability to excise 5fC and 5caC [164, 167]. TDG acts against thymine bases generated from 5mC deamination mispaired to G. The ability of TDG in rapidly excising fC than T from pairs with G [167] showed that the primary role of TDG might be in DNA demethylation rather than in deamination repair. Levels of TDG transcripts are extremely low in zygote and oocytes and are therefore not required for demethylation of the paternal pronucleus [163] but is highly essential for MET [148] during somitogenesis and organogenesis. Both TDG and SMUG1 can convert 5hmU to cytosine and are thought to act together with TET and AID/APOBEC [121, 158]. Such implications of their enzymatic activity were reported in a knockdown experiment that demonstrated that after TET-induced conversion of 5mC to 5hmC, AID/APOBEC mediates deamination of 5hmC to 5hmU and its further replacement occurs by an unmethylated cytosine through the BER pathway catalysed by TDG and SMUG1 [121, 158].

## 12. Nucleotide Excision Repair (NER) and Noncanonical Mismatch Repair (ncMMR) in DNA Demethylation

Barreto et al. [168] proposed that Gadd45a, a protein factor that can promote active demethylation in cultured mammalian cells, functions by NER as it requires NER endonuclease XPG, which directly binds to Gadd45a. However, another study in Gadd45a null mice could not confirm these findings as there was no observable increase in either global or locus-specific methylation [169]. The role of Gadd45a in active demethylation was also supported by a study which showed that active demethylation of the rRNA gene promoter is mediated by Gadd45a and the NER machinery. Gadd45b, a member of the Gadd45 family, was also observed to perform DNA demethylation at specific regulatory regions of Bdnf and Fgf1, two genes of adult neurogenesis [114]. It remains largely unknown as to which DNA demethylation pathways are stimulated by Gadd45 proteins. 5fC and 5caC may initiate transcription-coupled nucleotide excision repair (TC-NER) [170] as their presence in the template strand interferes with transcription [171, 172]. In addition to NER, ncMMR has also been implicated for the repair step of DNA demethylation. ncMMR process initiates at a nick in the DNA stand, removes mismatches, and replaces nucleotide patches from the nick to ~150 nucleotides beyond the mismatch site [173]. One recent study described nick-dependent repair by the ncMMR process that replaced 5mC nucleotides in DNA upon being triggered by uracil in the DNA [136]. ncMMR might be involved in AID-dependent demethylation as uracil plays a critical role in this process, but another report suggests its involvement in oxidation-dependent DNA demethylation [174]. The amount of research associating ncMMR and DNA demethylation is very minimal, and therefore, their involvement in the physiological and pathological circumstances has not yet been demonstrated.

## 13. Changes in DNA Methylome and Histone Methylation during AKI

Changes in DNA methylation were first reported by Pratt et al. [175] where they described the demethylation of a cytosine residue in the INF-*γ* response element within the compliment C3 promoter in response to cold ischemia in the rat kidney and additional demethylation during further warm reperfusion. The same authors also demonstrated that demethylation of the C3 promoter in rats with transplanted kidneys lasted for at least six months. The authors also proposed that during C3 promoter demethylation in ischemia-reperfusion injury (IRI) there occurs progressive oxidation of 5mC which was later proved in 2011 by the reports of Guo et al. [158]. In a study to examine the DNA methylation of gene promoters in urines of kidney transplant patients, Mehta et al. [176] reported that DNA methylation levels in the calcitonin (CALCA) promoter was higher in urines of kidney transplant recipients compared to those in healthy controls. They found that patients with acute tubular necrosis (proven by biopsy) compared to those that had acute rejection and slow graft function had higher DNA methylation levels. Their results strongly suggest the increase in 5mC levels during IRI. Endo et al. [177] reported that plasma levels of methylated DNA at Slc22a12 promoter region, a proximal tubular cells specific urate transporter, were significantly elevated after acute kidney cortex necrosis. In another study signifying the importance of epigenetic changes in renal kallikrein (KLK1) expression and susceptibility to AKI or recovery, Kang et al. [178] showed that promoter KLK1 CpG methylation was higher in blood than that in urine DNA. They studied the promoter CpG methylation of the KLK1 gene in blood and urine DNA from patients with established or incipient AKI and compared to healthy/nonhospital as well as ICU controls. They suggested that KLK1 methylation in blood DNA was significantly higher in established AKI than that in healthy controls, though KLK1 methylation in urine tended to be higher in AKI, directionally consistent with earlier/incipient but not later/established changes in KLK1 excretion in AKI.

The role of DNA demethylation in an AKI setting was recently demonstrated by Huang et al. [179] where they reported a global decrease in the 5hmC level in mouse with IRI-induced AKI while the global levels of 5mC remained unchanged. They found that these changes were a result of decreased levels of TET1, TET2, but not TET3 mRNA transcripts. They also identified lower 5hmC levels corresponding to Cxcl10 and Ifngr2 gene promoters with increased gene expression following IRI. However, they did not report the causal relationship between levels of 5hmC and gene expression. The same group in 2016 reported the relationship between gene expression and genomic distribution of 5hmC in the mouse kidney [180]. In their study, they profiled the DNA hydroxymethylome of the mouse kidney by hydroxymethylated DNA immunoprecipitation (hMeDIP-seq) and revealed that 5hmC is enriched in genic regions but depleted from intergenic regions. They further demonstrated that gene body enrichment of 5hmC is positively associated with the gene expression level in the mouse kidney. Also, during IRI-induced AKI, genes associated with IRI in the mouse kidney showed significantly higher 5hmC enrichment in their gene body regions when compared to those unchanged genes. Similar to that of DNA methylation studies, there are only few studies that have examined the effect of DNA demethylation in AKI. From the limited reports mentioned above, it is evident that AKI leads to changes in promoter DNA methylation with increase in 5mC levels in some genes and decrease in others. This is not surprising as there are several pathways regulating the activity of DNA methylation. Also, with advances in demethylation studies, it will be interesting to see if any of the several players of DNA demethylation emerges as a novel AKI biomarker.

The electrostatic interactions between the negatively charged DNA and the positively charged histone amino acid residues lead to a compact chromatin structure. Methylation of histones alters the transcription machinery by allowing the docking of chromatin modifiers. Although few, there are important studies reporting the implications of alterations in histone methylation (specifically in the lysine residues) in AKI. TNF-*α*, MCP-1, and HMGCR genes characteristically showed lysine 4 (K4) trimethylation (m3) on the histone H3 protein subunit in mouse models of AKI induced by IRI, endotoxin, UUO, and maleate [181–183]. The result of increase in H3K4m3 together with an increased expression of SET1 enzyme suggested that increased H3K4m3 levels are catalysed by this enzyme [184]. However, it was also shown that increased levels of H3K4m3 at these genes did not sustain in case of AKI induced by IRI [181]. In the study on urinary chromatin shed in patients with azotemia, Munshi et al. [185] found an increase in H3K4m3 levels in MCP-1 genes, therefore, highlighting the importance of urine as a source of histone epigenetic markers in AKI. In another study in patients with AKI, it was found that HMGCR activity got upregulated with increase in levels of H3K4m3 at exon 1 of the HMG-CoA reductase (HMGCR) gene [186]. As the existing data on the implications of histone methylation in AKI is scarce, it remains unclear whether any modulation of histone methylation would bring an alteration to the pathogenesis and regenerative responses after AKI.

## 14. Conclusion and Future Outlook

With recent advent of technological advancements in characterising the epigenome, the perspective of epigenetic research has drastically changed over the years. Essential parts of the mammalian development are the epigenetic modifications, and any disruption in this process results in detrimental cellular transformations. Recent advances have shed light on the possibility of a bidirectional dynamics in DNA methylation and demethylation that is regulated throughout the developmental stages of certain tissue types, mainly the brain [158, 187] to maintain a particular cellular epigenetic state. This hypothesis necessitates research to probe into the role, and coordination of methylation and demethylation marks in both healthy as well as disease state as much of this remains to be elucidated. The consequence of alterations in the methylome and the chromatin landscape has been extensively explored in cancer and neurological research; however, the same is not the case in AKI as renal epigenetic research is still at its infancy.

Despite the challenges, recent advances in epigenetic tools have made renal research more efficient [188]. High-throughput next generation DNA sequencing to examine a single nucleotide specific genome-wide DNA methylation pattern [91, 127], distinguishing 5hmC from 5mC in the genome by high-performance liquid chromatography with UV detection [189] or tandem mass spectrometry [190, 191], antibody enrichment of hydroxymethylated DNA or precipitation of modified 5hmC after biotinylation followed by microarray analysis [143, 145, 192–194], and quantification of 5mC and 5hmC at single base resolution by oxidative bisulfite sequencing [195] are few of many technological achievements that will advance our understanding of the epigenetics in the normal and pathological state of the kidney.

Understanding the *in vivo* physical phenomenon of de novo DNA methylation within a chromatin and its profound effect in AKI pathogenesis will be a good challenge to researchers. AKI is pathologically complex, and understanding how DNA methylation (a key regulator of transcriptional stability) integrates with other epigenetic modifications during the onset of AKI, its progression, and recovery of kidney after injury will enhance our knowledge on the state of methylome in renal injury. Research on active DNA demethylation is giving new impetus to studying 5hmC which is being considered as the “sixth” base. 5hmC is also being regarded as an epigenetic marker given its accumulation in certain tissues and cell types. Renal epigenetic research has so far seen very limited studies addressing 5hmC. Genome-wide studies have proposed 5hmC signatures as diagnostic biomarkers for human cancers [196]; however; only future studies will reveal if 5hmC can be considered as a worthy epigenetic marker of AKI or is just a simple intermediate of the DNA demethylation process in the kidney.

DNA methylation and demethylation are tightly regulated mechanisms, and there are several interesting questions that arise from their dynamicity, in general and in the context of AKI. However, here, we have enlisted only those that prompted from our understanding of the extent of epigenetic research in AKI. How do 5mC and 5hmC mark vary with different stages of AKI? Do all types of renal insults affect the epigenetic landscape during AKI? If so, are the results same and is it dependent on the extent of the injury? Will a dysregulated pathway or defective genes involved in DNA methylation lead to an aggravation of the injury? What controls the choice of the DNA demethylation pathway? Is the pathological implication due to the absence of TET enzymes similar to the reduced levels or lack of 5hmC? Can 5hmC be established as a renal epigenetic biomarker of AKI? Although a long shot, is it ever possible that renal epigenetic research might actually find the specific BER glycosylases required for physiological demethylation? Most of our knowledge on the epigenetic changes during AKI is limited due to lack of concrete experimental data. This questions every player and pathway of DNA methylation and demethylation in their role in AKI. It is well known that epigenetic modifications are reversible and therefore, therapy to modulate the epigenetic states is a promising option for not only AKI but other renal disorders as well. A quest towards answering the above questions and several other queries that would emerge in the way will yield new discoveries to diagnose and treat every stage of AKI.

## Figures and Tables

**Table 1:** Role of the methyl-binding domain protein family.

MBD family	Binding activity	Implication	Reference
MeCP2	Binds to methyl CpG with an adjacent stretch of AT-rich nucleotide	Repression of transcriptional activity	[80]
MBD1	Binds to methyl CpG via methyl-binding domain	Gene silencing during DNA replication	[75]
MBD2	Binds to methyl CpG	Repression of transcriptional activity	[85]
MBD3	Low binding/incapable of binding to methyl CpG	Subunit of NuRD corepressor complex and acts as a transcriptional repressor	[86]
MBD4	Binds to methyl CpG and T:G mismatches at methyl CpG sites	Catalyses the removal of T and U paired with G within CpG sites	[87]
